# OPPORTUNITIES FOR USING HIGH-FREQUENCY ASSESSMENT METHODS TO ADVANCE THE STUDY OF COGNITIVE CHANGE

**DOI:** 10.1093/geroni/igad104.2042

**Published:** 2023-12-21

**Authors:** Martin Sliwinski

**Affiliations:** Pennsylvania State University, State College, Pennsylvania, United States

## Abstract

Embedding mobile cognitive assessments into high frequency assessment (HFA) designs, such as ecological momentary assessments or measurement bursts, affords novel opportunities for the study of both short-term and long-term change. By collecting frequent measurements across short periods of time, it is possible to model short-term within-person trends and fluctuations in cognitive performance, and to relate those features to changes over longer time periods, clinical phenotypes, and neurodegenerative processes. In this presentation, we will review contemporary approaches for HFA of cognitive function and evaluate prior studies utilizing these approaches to examine the short-term coupling among naturally occurring variations in psychosocial variables such as social activity and stress with cognitive function. The challenges and potential for using high frequency mobile cognitive assessments to discover and characterize proximal contextual influences on cognitive functioning and performance will be discussed.

